# Organometal Halide Perovskite‐Based Photoelectrochemical Module Systems for Scalable Unassisted Solar Water Splitting

**DOI:** 10.1002/advs.202303106

**Published:** 2023-09-26

**Authors:** Hojoong Choi, Sehun Seo, Chang Jae Yoon, Jae‐Bin Ahn, Chan‐Sol Kim, Yoonsung Jung, Yejoon Kim, Francesca M. Toma, Heejoo Kim, Sanghan Lee

**Affiliations:** ^1^ School of Materials Science and Engineering Gwangju Institute of Science and Technology Gwangju 61005 Republic of Korea; ^2^ Chemical Sciences Division Lawrence Berkeley National Laboratory 1 Cyclotron Road Berkeley CA 94720 USA; ^3^ Liquid Sunlight Alliance Lawrence Berkeley National Laboratory 1 Cyclotron Road Berkeley CA 94720 USA; ^4^ Institute of Functional Materials for Sustainability Helmholtz‐Zentrum Hereon Kantstraße 55 14513 Teltow Germany; ^5^ Research Institute for Solar and Sustainable Energies Gwangju Institute of Science and Technology Gwangju 61005 Republic of Korea; ^6^ Graduate School of Energy Convergence Institute of Integrated Technology, Gwangju Institute of Science and Technology Gwangju 61005 Republic of Korea; ^7^ Research Center for Innovative Energy and Carbon Optimized Synthesis for Chemicals (Inn‐ECOSysChem) Gwangju Institute of Science and Technology Gwangju 61005 Republic of Korea

**Keywords:** organometal halide perovskite, photoelectrochemical water splitting, unassisted solar water splitting, scalable, module

## Abstract

Despite achievements in the remarkable photoelectrochemical (PEC) performance of photoelectrodes based on organometal halide perovskites (OHPs), the scaling up of small‐scale OHP‐based PEC systems to large‐scale systems remains a great challenge for their practical application in solar water splitting. Significant resistive losses and intrinsic defects are major obstacles to the scaling up of OHP‐based PEC systems, leading to the PEC performance degradation of large‐scale OHP photoelectrodes. Herein, a scalable design of the OHP‐based PEC systems by modularization of the optimized OHP photoelectrodes exhibiting a high solar‐to‐hydrogen conversion efficiency of 10.4% is suggested. As a proof‐of‐concept, the OHP‐based PEC module achieves an optimal PEC performance by avoiding major obstacles in the scaling up of the OHP photoelectrodes. The constructed OHP module is composed of a total of 16 OHP photoelectrodes, and a photocurrent of 11.52 mA is achieved under natural sunlight without external bias. The successful operation of unassisted solar water splitting using the OHP module without external bias can provide insights into the design of scalable OHP‐based PEC systems for future practical application and commercialization.

## Graphical abstract

Organometal halide perovskite (OHP)‐based photoelectrochemical (PEC) module is introduced as a new approach for scalable design of OHP‐based PEC system and characterized under outdoor conditions for real‐world verification in this study. The 4 cm^2^ active area of OHP PEC module exhibits a photocurrent of 11.52 mA without external bias under natural sunlight.

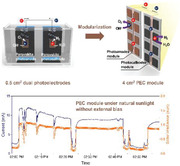

## 1 Introduction

As an inexhaustible energy source, solar energy is regarded as a potential candidate for renewable energy sources to replace fossil fuels.^[^ ^1^ ^]^ However, the conversion of solar energy into a transportable and storable fuel, hydrogen (H_2_), is required to manage the intermittent nature of solar energy supply.^[^ ^2^ ^]^ Solar water splitting by photoelectrochemical (PEC) systems provides great potential to achieve sustainable hydrogen (H_2_) production.^[^ ^3^ ^]^ For commercially viable PEC systems, the solar‐to‐hydrogen (STH) conversion efficiency has been suggested to be 10%.^[^ ^3^, ^4^, ^5^ ^]^ Accordingly, various studies have focused on the development of suitable materials for efficient photoelectrodes to achieve an STH conversion efficiency of 10% using PEC systems. To date, various semiconductors such as metal‐oxides,^[^ ^6^, ^7^, ^8^ ^]^ chalcogenides,^[^ ^9^, ^10^, ^11^ ^]^ and silicon‐based photoelectrodes^[^ ^12^ ^]^ have been developed for PEC water splitting. However, the PEC performance of the photoelectrodes fabricated with these materials is still insufficient to achieve an STH conversion efficiency of 10%. Several candidates, especially III–V compound‐based PEC systems, have achieved an STH conversion efficiency over 10%,^[^ ^13^, ^14^ ^]^ but unfortunately, the high cost of III–V compounds still greatly restricts the practical application of PEC systems for solar water splitting.^[^ ^15^ ^]^ Thus, the development of both cost‐effective and efficient photoelectrodes is urgently required for sustainable solar H_2_ production through the realization of the practical application of PEC systems.

Organometal halide perovskites (OHPs) have become breakthrough materials for the development of efficient and cost‐effective photoelectrodes for solar water splitting due to their excellent optoelectronic properties and potential for low‐temperature solution processability.^[^ ^16^, ^17^ ^]^ With these advantages, great efforts have been made to construct OHP‐based PEC systems for unassisted solar water splitting without external bias.^[^ ^18^, ^19^ ^]^ However, conventional OHP‐based tandem PEC cells connected with BiVO_4_ exhibited a low STH conversion efficiency of 1.26% due to the relatively low PEC performance of the BiVO_4_ photoanodes.^[^ ^19^ ^]^ For the construction of OHP‐based PEC systems with the highest efficiency, that is, more than STH conversion efficiency of 10%, it is essential to develop PEC systems with OHP dual photoelectrodes composed of OHP photoanodes and photocathodes. Accordingly, OHP dual photoelectrodes for unassisted solar water splitting, which have exhibited remarkable efficiency, have been reported.^[^ ^20^ ^]^

Although remarkable advances have been achieved in OHP‐based PEC systems by connecting the OHP photoanodes and photocathodes, their scalability remains one of the main issues limiting their practical application. The scalability of OHP‐based PEC systems, especially OHP dual photoelectrodes, is quite limited due to the significant resistive losses and intrinsic defects caused by an increase in the active area of the OHP photovoltaic (PV) cells.^[^ ^18^, ^21^, ^22^, ^23^ ^]^ For these reasons, the active area of the OHP‐based photoelectrodes to achieve the optimal efficiency is standardized on a small‐scale. Thus, to date, the unassisted solar water splitting by OHP dual photoelectrodes has been reported only on a small‐scale with an active area of less than 1 cm^2^.^[^ ^20^ ^]^ For OHP dual photoelectrodes to advance beyond the laboratory‐scale as large‐scale PEC systems suitable for the practical application of solar water splitting, their rational design should be considered.

Herein, we report scalable OHP‐based PEC module systems (OHP module) to drive unassisted solar water splitting without external bias. To achieve the high PEC performance of the cells required for the modules, the catalyst‐integrated encapsulation layers and electrical contact materials between them are optimized. Using the optimized small‐scale OHP‐based dual photoelectrodes composed of NiFe layered double hydroxides (LDH)/Ni foil/OHP photoanodes and NiMo/Ni foil/OHP photocathodes, we achieve a high photocurrent density of 8.75 mA cm^−2^ and STH conversion efficiency of 10.4% without external bias. In addition to the remarkable STH conversion efficiency over 10%, the long‐term PEC stability of the OHP‐based dual photoelectrodes achieved in this study provides the opportunity to realize cost‐effective, efficient, and stable solar H_2_ production. For the next step, scaling up of the optimized small‐scale OHP photoelectrodes while maintaining the optimal PEC performance, we consider the rational design of OHP‐based PEC systems. To this end, we demonstrate an OHP‐based PEC module in which the OHP photoelectrodes are connected in arrays of cells. Because the active area of ​​OHP PV cells for optimal efficiency is standardized, we report an OHP module to show the optimal efficiency by connecting standardized OHP photoelectrode arrays with high PEC performance. As a proof‐of‐concept, the operation of unassisted solar water splitting with the OHP module is successfully demonstrated under natural sunlight conditions, thus providing the potential of a new approach to scalable OHP‐based PEC systems.

## 2 Results and Discussion

To fabricate efficient and stable OHP‐based dual photoelectrodes, the following factors should be considered: 1) OHP PV cells with high power conversion efficiency (PCE), 2) long‐term stable OHP PV cells, 3) metal foil encapsulation layers to prevent water molecules, 4) integration of catalysts with high catalytic activity, and 5) appropriate electrical contact materials between the OHP PV cells and encapsulation layers. In this study, we fabricated and characterized efficient and stable OHP‐based dual photoelectrodes in consideration of the above‐mentioned factors.

**Figure 1:**
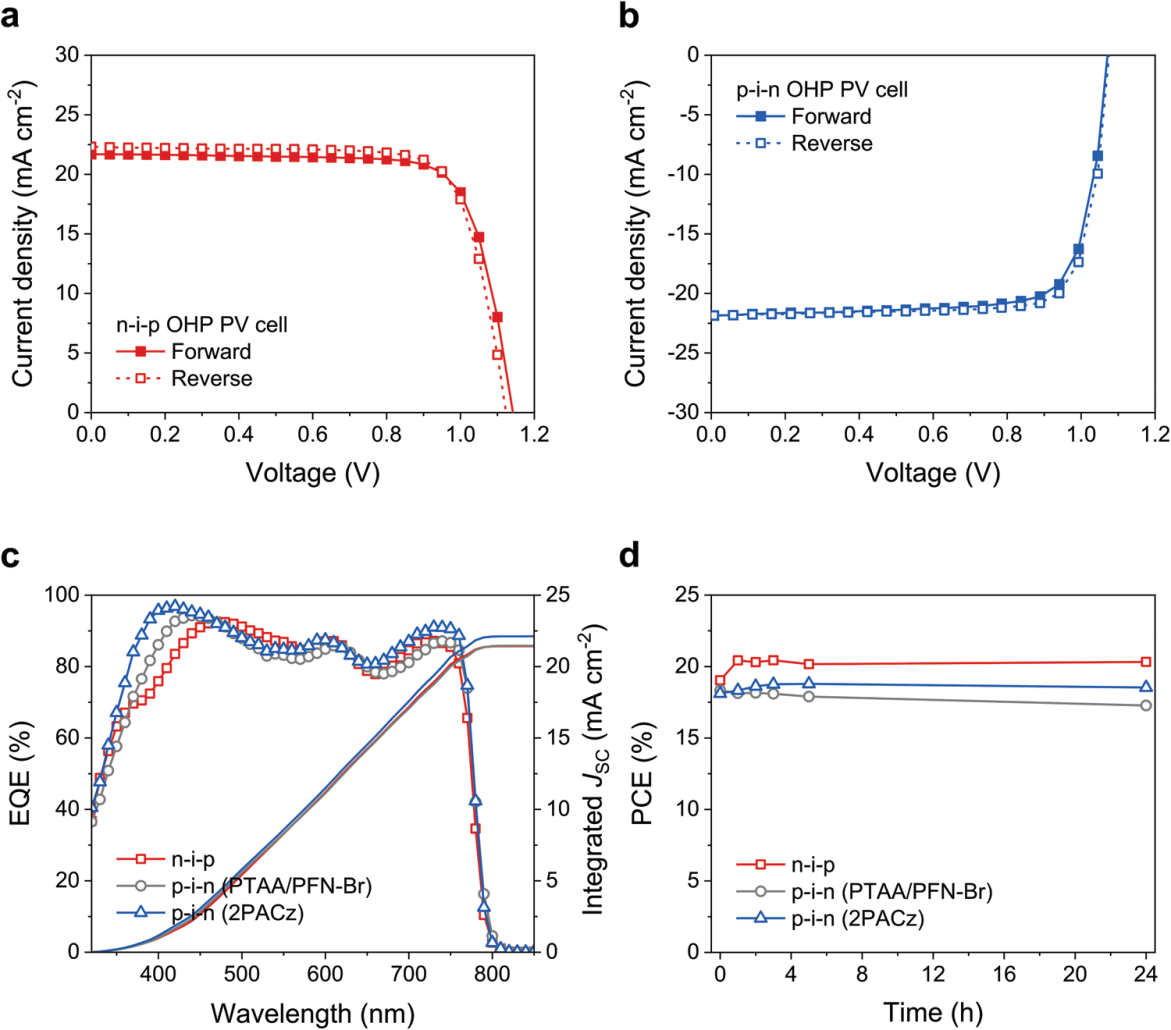
PV performance of the n–i–p and p–i–n OHP PV cells. Representative *J*−*V* curves of the a) n–i–p and b) p–i–n OHP PV cells. c) EQE spectra and corresponding integrated *J*_SC_ of the n–i–p and p–i–n OHP PV cells. d) Stability of the n–i–p and p–i–n OHP PV cells.

The PV performance of the OHP PV cells is presented in **Figure** **1**a,b with the representative current density–voltage (*J*–*V*) curves. The best performing n–i–p OHP PV cell exhibited a high PCE of 19.15% with a short‐circuit current density (*J*_SC_) of 21.69 mA cm^−2^, open‐circuit voltage (*V*_OC_) of 1.14 V, and fill factor (FF) of 77% (Figure 1a). The best performing p–i–n OHP PV cell also exhibited a high PCE of 18.81% with a *J*_SC_ of 21.85 mA cm^−2^, *V*_OC_ of 1.08 V, and FF of 80% (Figure 1b). In our case, we employed [2‐(9*H*‐carbazol‐9‐yl)ethyl]phosphonic acid (2PACz) as hole transport layers (HTLs) in the p–i–n OHP PV cells instead of multi‐layered HTLs; poly[bis(4‐phenyl)(2,4,6‐trimethylphenyl)amine]/poly[9,9‐bis(3′‐(*N*,*N*‐dimethyl)‐*N*‐ethylammonium‐propyl‐2,7‐fluorene)‐*alt*‐2,7‐(9,9‐dioctylfluorene)]dibromide (PTAA/PFN‐Br) HTLs. 2PACz has deeper highest occupied molecular orbitals compared with PTAA, reducing the energetic barrier at the OHP/HTL interface.^[^ ^24^ ^]^ Therefore, the p–i–n OHP PV cells using 2PACz HTLs exhibited higher PCE compared with the device using PTAA/PFN‐Br HTLs. The statistical PCE distribution confirms that a higher average PCE and reproducibility are demonstrated with 2PACz than PTAA/PFN‐Br HTLs for the p–i–n OHP PV cells as shown in Figure S1 (Supporting Information). The corresponding results are summarized in Table S1 (Supporting Information). The external quantum efficiency (EQE) spectra and the corresponding integrated *J*_SC_ of the OHP PV cells are presented in Figure 1c. Both the n–i–p and p–i–n OHP PV cells exhibited a high EQE in the wavelength range of 350−750 nm (Figure 1c). It is noticeable that the p–i–n OHP PV cell using 2PACz HTL exhibited a higher EQE than that with PTAA/PFN‐Br HTL. The integrated *J*_SC_ of the n–i–p, p–i–n (PTAA/PFN‐Br), and p–i–n (2PACz) are 21.43, 21.46, and 22.12 mA cm^−2^, respectively, which are similar to the *J*_SC_ obtained from *J*–*V* curves (Figure 1c).

Instability factors, such as water molecules and light illumination, cause severe degradation during operation of the OHP PV cells.^[^ ^25^, ^26^ ^]^ In general, the OHP photoelectrodes suffer from more severe instability factors, that is, high humidity and environment in which charge traps can easily accumulate in addition to light illumination during the operation of PEC water splitting.^[^ ^27^, ^28^ ^]^ For these reasons, the fabrication of OHP photoelectrodes using OHP PV cells with high stability is desired. As shown in Figure 1d, both nonencapsulated n–i–p and p–i–n OHP PV cells exhibited high stability. For evaluation of stability, the OHP PV cells were kept under dark and ambient conditions (25 °C/25% relative humidity (RH)). For the p–i–n OHP PV cells, compared with the devices using PTAA/PFN‐Br HTLs, higher stability was exhibited with the device using 2PACz HTLs (Figure 1d). In addition, the stability of OHP PV cell was evaluated after kept under 25 °C/85% RH conditions. As shown in Figure S2 (Supporting Information), the p–i–n OHP PV cell with 2PACz HTLs exhibited high stability under 25 °C/85% RH conditions. Therefore, these two evaluations of the stability clearly suggest that the p–i–n OHP PV cells with 2PACz HTLs are suitable OHP photocathodes for OHP‐based PEC systems.

To prevent the penetration of water molecules to the OHP layers and to simultaneously promote the reaction kinetics of PEC water splitting, we combined catalyst‐integrated Ni foil encapsulation layers with the OHP PV cells. For the oxygen evolution reaction (OER) catalysts of the OHP photoanodes, NiFe LDH, which is one of the representative OER catalysts with a high OER catalytic activity in alkaline electrolytes, was selected to be integrated with the Ni foils. For the hydrogen evolution reaction (HER) catalysts of the OHP photocathodes, a more careful selection of the HER catalysts to be integrated with Ni foils is required to realize cost‐effective, efficient, and stable unassisted solar water splitting with OHP dual photoelectrodes. In general, it is well‐known that higher HER catalytic activity is exhibited in acidic electrolytes.^[^ ^29^ ^]^ Note that the previously reported OHP photocathodes showing high PEC performance were evaluated in acidic electrolytes.^[^ ^27^, ^28^ ^]^ Hence, for the HER catalysts of the OHP photocathodes to be driven in alkaline electrolytes, we considered Ni‐based alloys, which are known to show high HER catalytic activity in alkaline electrolytes.^[^ ^30^ ^]^ Among them, NiMo catalysts have shown high HER catalytic activity comparable to that of Pt in alkaline electrolytes.^[^ ^30^, ^31^ ^]^ For these reasons, in this study, the NiFe LDH and NiMo were integrated with Ni foils as OER and HER catalysts, respectively, and subsequently combined with the OHP PV cells to fabricate the OHP‐based dual photoelectrodes to be driven in alkaline electrolytes.

**Figure 2:**
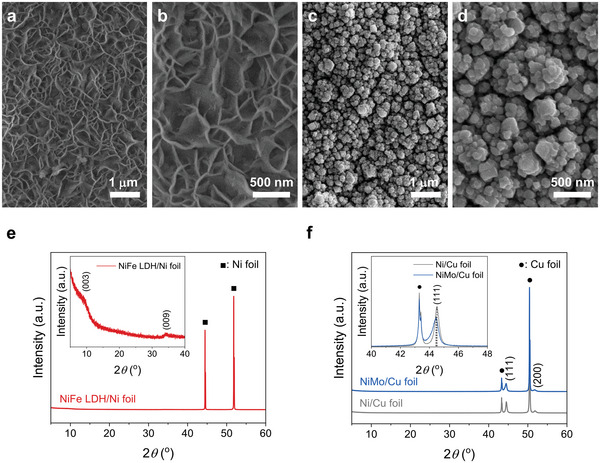
Morphological and structural characteristics of catalyst‐integrated encapsulation layers. FESEM images of the a,b) NiFe LDH/Ni foil and c,d) NiMo/Ni foil with different magnifications. XRD patterns of the e) NiFe LDH/Ni foil, f) Ni/Cu foil, and NiMo/Cu foil. The insets show expanded regions of the XRD patterns.

The NiFe LDH/Ni foils and NiMo/Ni foils were fabricated using the facile electrodeposition methods.^[^ ^31^, ^32^ ^]^ The morphologies of the catalysts were investigated using field emission scanning electron microscopy (FESEM). **Figure** **2**a,b shows the morphology of the nanosheet arrays, which is the typical morphology of LDHs.^[^ ^32^ ^]^ On the other hand, the porous nanoparticulate morphology, which is similar to the previously reported morphologies of NiMo catalysts fabricated using electrodeposition, was observed as shown in Figure 2c,d.^[^ ^31^ ^]^ The structural characteristics of the NiFe LDH/Ni foil and NiMo/Ni foil were investigated by X‐ray diffraction (XRD) patterns. As shown in Figure 2e, the NiFe LDH/Ni foil showed no significant difference in the XRD peaks, indicating the amorphous nature of the electrodeposited NiFe LDH.^[^ ^32^, ^33^ ^]^ However, the weak diffraction peaks of the (003) and (009) planes of NiFe LDH also indicate the certain formation of NiFe LDH with low crystallinity via electrodeposition as shown in the inset of Figure 2e.^[^ ^32^ ^]^ Meanwhile, the XRD pattern of NiMo/Ni foil shows no significant XRD peaks because the peaks of NiMo are overlapped with those of the Ni foil (Figure S3, Supporting Information). Thus, the XRD pattern of NiMo/Cu foil was analyzed after the electrodeposition of NiMo on Cu foil to investigate the phase of NiMo. As shown in Figure 2f, the diffraction peaks of the (111) and (200) planes of NiMo are observed.^[^ ^31^, ^34^ ^]^ To identify the Mo atom in the NiMo/Cu foil, we analyzed the XRD pattern after the electrodeposition of Ni without the Mo precursor on Cu foil. Although the NiMo/Cu foil showed similar XRD peaks to the Ni/Cu foil as shown in Figure 2f, the XRD peaks were slightly shifted compared to those of the Ni/Cu foil. The inset of Figure 2f shows the shifted diffraction peak of the (111) plane of NiMo compared to that of Ni. This shift of the XRD peaks are attributed to the lattice expansion caused by the substitution of larger Mo atoms in the Ni lattice.^[^ ^34^, ^35^ ^]^ To verify the morphological and structural characteristics of NiFe LDH and NiMo in detail, we performed transmission electron microscopy (TEM) analysis. Similar to the FESEM images of NiFe LDH (Figure 2a,b), nanosheet morphology was observed in the TEM image of NiFe LDH (Figure S4a, Supporting Information). The selective area electron diffraction (SAED) pattern of NiFe LDH (Figure S4b, Supporting Information) indicates the low crystallinity of electrodeposited NiFe LDH consistent with the XRD pattern (Figure 2e). High‐angle annular dark‐field scanning TEM (HAADF‐STEM) image of NiFe LDH (Figure S4c, Supporting Information) and corresponding energy‐dispersive spectroscopy (EDS) mapping results show that Ni and Fe elements are uniformly distributed (Figure S4d,e, Supporting Information). The TEM was also performed on the NiMo. As shown in the TEM image, NiMo was successfully formed on Ni foil using electrodeposition (Figure S5a, Supporting Information). In addition, the nanoparticulate morphology of NiMo was observed in the TEM image (Figure S5a, Supporting Information) as in the FESEM images (Figure 2c,d). The SAED pattern of NiMo (Figure S5b, Supporting Information) indicates the polycrystalline NiMo was formed. HAADF‐STEM image of NiMo/Ni foil shows the thickness of NiMo is about 2.4 µm (Figure S5c, Supporting Information) and corresponding EDS mapping results of NiMo/Ni foil (Figure S5d,e, Supporting Information) confirm that the Mo element exists in electrodeposited NiMo, while the Ni element is observed in both NiMo and Ni foil. The chemical states of the NiFe LDH/Ni foil and NiMo/Ni foil were investigated using X‐ray photoelectron spectroscopy (XPS). For NiFe LDH, the high‐resolution XPS spectra of Ni 2p shown in Figure S6a (Supporting Information), presents peaks at 872.9 and 855.3 eV, along with their satellite peaks at 879.4 and 861.1 eV, respectively, confirming the existence of Ni^2+^ in NiFe LDH. In the Fe 2p spectra (Figure S6b, Supporting Information), the peaks at 724.7 and 711.5 eV, along with their small satellite peaks around 733.6 and 718.4 eV, respectively, also confirm the existence of Fe^2+^ in NiFe LDH.^[^ ^32^, ^36^ ^]^ For NiMo, the high‐resolution XPS spectra of Ni 2p shown in Figure S6c (Supporting Information), presents the peaks at 869.3 and 852.1 eV, which confirm the existence of metallic Ni (Ni^0^) in NiMo. In addition, the peaks at 873.3 and 855.5 eV, along with their satellite peaks at 879.9 and 860.9 eV, respectively, in the Ni 2p spectra confirm the existence of Ni^2+^ in NiMo (Figure S6c, Supporting Information). In the Mo 3d spectra (Figure S6d, Supporting Information), the peak at 227.4 eV corresponds to the metallic Mo (Mo^0^) in NiMo. In addition, the peaks at 235.2 and 232 eV, along with the peak at 230.7 eV, confirm the existence of Mo^6+^ and Mo^5+^ in NiMo, respectively (Figure S6d, Supporting Information).^[^ ^31^ ^]^ These results indicate the successful preparation of the NiFe LDH and NiMo catalysts on Ni foil using electrodeposition.

Before combining the catalyst‐integrated encapsulation layers with the OHP PV cells, we investigated the catalytic activities of the NiFe LDH/Ni foils and NiMo/Ni foils were investigated in 1 m KOH electrolytes, using a three‐electrode configuration. Figure S7a (Supporting Information) shows the representative cyclic voltammetry (CV) curves of the NiFe LDH/Ni foils with different molar ratios of the precursor. The NiFe LDH/Ni foils exhibited high OER catalytic activities with a low overpotential, as shown in Figure S7a (Supporting Information), regardless of the molar ratio of the precursor. However, among them, the NiFe LDH/Ni foil with a 3:1 molar ratio of the precursor exhibited the highest OER catalytic activity. Meanwhile, Figure S7b (Supporting Information) shows the representative CV curves of the NiMo/Ni foils electrodeposited at different current densities. The NiMo/Ni foils electrodeposited at high current densities of −60, −80, and −100 mA cm^−2^ exhibited high HER catalytic activities with a low overpotential in the alkaline electrolytes, as shown in Figure S7b (Supporting Information). On the other hand, the NiMo/Ni foils electrodeposited at low current densities of −20 and −40 mA cm^−2^ exhibited relatively low HER catalytic activities (Figure S7b, Supporting Information). The improved HER catalytic activities of the NiMo/Ni foils electrodeposited at high current densities were attributed to the widened gap between the NiMo nanoparticles due to the HER during electrodeposition at high current densities, which serves as an ion diffusion channel.^[^ ^37^ ^]^ The electrochemical (EC) stabilities of the NiFe LDH/Ni foil and NiMo/Ni foil were measured by chronopotentiometric measurements. As shown in Figure S7c (Supporting Information), the NiFe LDH/Ni foil exhibited stable OER catalytic activity at 20 mA cm^−2^ without significant degradation. The NiMo/Ni foil also exhibited stable HER catalytic activity at −20 mA cm^−2^, as shown in Figure S7d (Supporting Information). These results indicate that the catalysts integrated with Ni foils can effectively improve the performance of the OHP dual photoelectrodes for unassisted solar water splitting in alkaline electrolytes.

**Figure 3:**
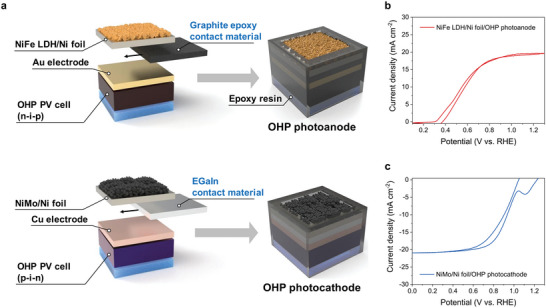
Schematic representations and PEC performance of the OHP photoelectrodes. a) Fabrication process of the vertically stacked NiFe LDH/Ni foil/OHP photoanode and NiMo/Ni foil/OHP photocathode. Representative CV curves of the b) NiFe LDH/Ni foil/OHP photoanode, and c) NiMo/Ni foil/OHP photocathode.

The NiFe LDH/Ni foils and NiMo/Ni foils served as encapsulation layers to prevent the penetration of water molecules into the OHP layers and simultaneously improve the OER and HER catalytic activities, respectively, thereby promoting the reaction kinetics of PEC water splitting. In this respect, the OHP PV cells, catalyst‐integrated encapsulation layers, and their electrical contact materials were vertically stacked as shown in the schematic representations of the OHP photoanode and photocathode (**Figure** **3**a). As shown in Figure 3a, the n–i–p OHP PV cells and NiFe LDH/Ni foils were combined by graphite epoxy. Although the In–Ga eutectic alloy (EGaIn), which has been used in previous reports, is a suitable contact material for minimizing charge loss between the encapsulation and OHP layers, the Ga in EGaIn can chemically react with the Au electrodes.^[^ ^38^ ^]^ Considering this aspect, we employed graphite epoxy for the fabrication of the OHP photoanodes to avoid the dissolution of the Au electrodes of n–i–p OHP PV cells.^[^ ^19^ ^]^ On the other hand, the p–i–n OHP PV cells and NiMo/Ni foils were combined by EGaIn as in previous reports (Figure 3a).^[^ ^27^, ^28^ ^]^

We measured the PEC performance of the fabricated OHP photoanodes and photocathodes in 1 m KOH electrolytes using a three‐electrode configuration under AM 1.5 G illumination (100 mW cm^−2^). The representative CV curve of the NiFe LDH/Ni foil/OHP photoanode is shown in Figure 3b. As shown in Figure 3b, the NiFe LDH/Ni foil/OHP photoanode exhibited a photocurrent density of 19.7 mA cm^−2^ at 1.23 V versus reversible hydrogen evolution (vs. RHE) and an onset potential around 0.4 V versus RHE, which are comparable to those of previously reported OHP photoanode.^[^ ^39^ ^]^ Considering that still low PEC performance of BiVO_4_, which is a representative photoanode for PEC water splitting,^[^ ^40^ ^]^ a high PEC performance achieved by the NiFe LDH/Ni foil/OHP photoanode in this study is a more suitable photoanode for unassisted solar water splitting. The PEC performance of the OHP photocathode was also investigated. As shown in the CV curve (Figure 3c), the NiMo/Ni foil/OHP photocathode exhibited a photocurrent density of −21 mA cm^−2^ at 0 V versus RHE and an onset potential around 1.03 V vs.versus RHE. Note that the PEC performance of the NiMo/Ni foil/OHP photocathode is higher than that of OHP photocathode integrated with similar earth‐abundant catalyst in an alkaline electrolyte (CoP/Sn/OHP photocathode with −12.4 mA cm^−2^ and 1 V versus RHE, respectively).^[^ ^20^ ^]^ In this study, the onset potentials of OHP photoanodes and photocathodes were determined as the potential when the photocurrent of OHP photoelectrodes in the backward sweeps of CV curves reached 1 and −1 mA cm^−2^, respectively. More interestingly, the NiFe LDH/Ni foil/OHP photoanode and NiMo/Ni foil/OHP photocathode exhibited long‐term PEC stability over 24 h, as shown in Figure S8a,b (Supporting Information), respectively. In addition, the O_2_ and H_2_ generated at the OHP photoelectrodes and corresponding Faradaic efficiencies (*η*_F_) were evaluated. As shown in Figure S9a (Supporting Information), a total O_2_ of 85 µmol was generated at the NiFe LDH/Ni foil/OHP photoanode after 2 h and the average *η*_F_ for O_2_ evolution was 95.6%. Likewise, a total H_2_ of 193 µmol was generated at the NiMo/Ni foil/OHP photocathode after 2 h, and the average *η*_F_ for H_2_ evolution was 96.6%, as shown in Figure S9b (Supporting Information). As both the OHP photoanode and photocathode in this study exhibited excellent PEC performance, a higher photocurrent density and STH conversion efficiency of unassisted solar water splitting using dual OHP photoelectrodes compared with conventional BiVO_4_−OHP PEC tandem cells^[^ ^18^, ^19^ ^]^ and previously reported OHP dual photoelectrodes are expected.^[^ ^20^ ^]^

**Figure 4:**
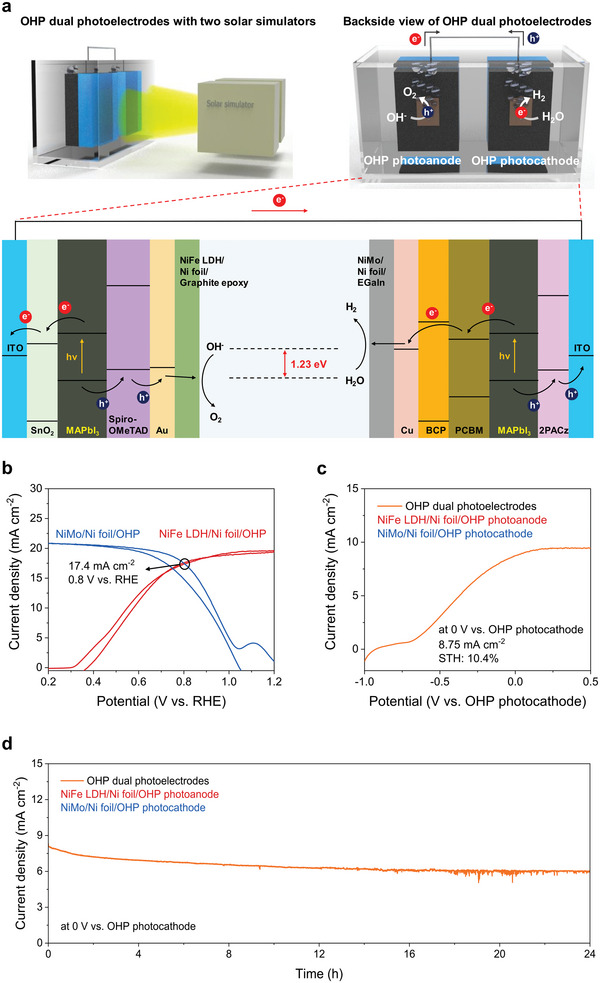
PEC performance of the OHP dual photoelectrodes for unassisted solar water splitting. a) Schematic representation of the OHP‐based PEC systems for unassisted solar water splitting and the corresponding band alignment. b) Combined CV curves of the OHP photoanode and photocathode for expectation of the operating point of the dual photoelectrodes. c) Representative LSV curve of the OHP dual photoelectrodes using a two‐electrode configuration. d) Chronoamperometric measurement for evaluation of the PEC stability of the OHP dual photoelectrodes without external bias.

To measure the PEC performance of the OHP dual photoelectrodes without external bias, the OHP photoanode and photocathode were connected in a “parallel illumination” configuration and the PEC performance was measured by two solar simulators (**Figure** **4**a).^[^ ^41^ ^]^ As we constructed the “parallel illumination” configuration, a total active area of 0.5 cm^2^ was used for evaluation of unassisted solar water splitting using the OHP dual photoelectrodes.^[^ ^42^ ^]^ The energy level diagram of the OHP dual photoelectrodes under light illumination is presented in Figure 4a. The operating point of the OHP dual photoelectrodes for unassisted solar water splitting is expected to be 17.4 mA cm^−2^ at 0.8 V versus RHE, as shown in Figure 4b, which corresponds to the intersection point of the CV curves of the OHP photoanode and photocathode shown in Figure 3b,c. We measured the PEC performance of the OHP dual photoelectrodes in 1 m KOH electrolytes using a two‐electrode configuration under an AM 1.5 G illumination (100 mW cm^−2^). The configuration of the OHP dual photoelectrodes during the PEC performance measurements is shown in the photograph in Figure S10 (Supporting Information). The representative linear sweep voltammetry (LSV) curve in Figure 4c shows that the OHP dual photoelectrodes exhibited a high photocurrent density of 8.75 mA cm^−2^ at 0 V versus OHP photocathode (i.e., unassisted condition), which corresponded to an STH conversion efficiency of 10.4% assuming a 96.6% of *η*_F_ for H_2_ evolution. Moreover, a photocurrent density of 72.2% of the OHP dual photoelectrodes without external bias was maintained after 24 h without breakdown of the photocurrent density as shown in Figure 4d. A photograph of the OHP photoelectrodes after PEC stability measurements, shown in Figure S11 (Supporting Information), indicates that no observable damage for them. Note that a breakdown or severe decrease in the photocurrent density of some previously reported OHP‐based PEC tandem cells was observed even before 24 h of continuous illumination without external bias.^[^ ^18^, ^19^ ^]^ Because the catalyst‐integrated Ni foil encapsulation layers provide efficient protection of the OHP layer and promote the reaction kinetics of PEC water splitting, high STH conversion efficiency and long‐term PEC stability of the OHP dual photoelectrodes without external bias were achieved in this study.

The STH conversion efficiency of 10.4% achieved with the OHP dual photoelectrodes in this study is comparable to that of previously reported state‐of‐the‐art PEC systems for unassisted solar water splitting. Note that the low STH conversion efficiency of the Cu(In,Ga)Se_2_ (CIGS)^[^ ^9^ ^]^ and Cu_2_ZnSnS_4_ (CZTS)‐based PEC tandem cells,^[^ ^11^ ^]^ which were connected with BiVO_4_ photoanodes despite their high half‐cell PEC performance of photocathodes (half‐cell efficiencies of 12.5% and 7.27%, respectively). These results clearly suggest that the low PEC performance of BiVO_4_ limits the overall STH conversion efficiency of unassisted solar water splitting,^[^ ^9^ ^]^ and thus the development of photoanodes with higher PEC performance is essential. The OHP‐based PEC tandem cells also suffered from a low STH conversion efficiency due to the low PEC performance of the BiVO_4_ photoanodes connected to the OHP photocathodes.^[^ ^18^, ^19^ ^]^ More interestingly, the high STH conversion efficiency of the OHP dual photoelectrodes in this study was achieved through the solution process, which enables cost‐effective, facile low‐temperature fabrication and use of earth‐abundant catalysts. Considering that an approach to next‐generation solar energy technology is to obtain cost advantages while increasing efficiency,^[^ ^43^ ^]^ it is quite remarkable that a high STH conversion efficiency was achieved with the OHP dual photoelectrodes in this study. For example, the PEC systems composed of GaAs‐based dual photoelectrodes exhibited a high STH conversion efficiency of 12.7%.^[^ ^13^ ^]^ However, these PEC systems are not suitable for practical unassisted solar water splitting due to the high cost of the III–V compounds and complicated fabrication process.^[^ ^15^ ^]^ For these reasons, these emerging OHP‐based dual photoelectrodes can be potential candidates for practical unassisted solar water splitting because their cost‐effective, facile fabrication process, and high STH conversion efficiency over 10%, which are essential requirements for commercial PEC systems.

Unfortunately, the great potentials of the OHP‐based dual photoelectrodes were only verified in a laboratory environment with a small active area of less than 1 cm^2^ and under continuous simulated 1 sun illumination.^[^ ^20^ ^]^ Therefore, several advances, such as scaling‐up of the active area and real‐world verification, are essential for the practical application of OHP dual photoelectrodes. However, these advances, especially the scaling‐up of the active area of OHP‐based photoelectrodes, are significantly limited. As increasing the active area, the resistive losses as well as the intrinsic defects in OHP layers such as pinholes and interfacial defects increase, thereby reducing the PCE of the OHP PV cells.^[^ ^18^, ^21^ ^−^ ^23^ ^]^ To verify the resistive loss depending on the active areas of OHP PV cells, we fabricated and characterized the OHP PV cells with various active areas (0.04–1 cm^2^). The active areas of fabricated OHP PV cells were determined by varying the areas of top electrodes after fabrication of OHP PV cells with process as the conventional OHP PV cells in this study. Both n–i–p and p–i–n OHP PV cells exhibited lower PV performance as the active area increased (Figure S12a,b, Supporting Information, respectively). These results can be attributed to the intrinsic defects of the OHP layer, such as pinholes and interfacial defects, as well as increased resistive losses. This efficiency loss in large‐scale devices is also unavoidable in the case of the OHP‐based photoelectrodes.^[^ ^18^ ^]^ Furthermore, the PEC performance degradation due to increasing the active area of the half‐cell OHP photoelectrodes is overlapped at each of the OHP photoanodes and photocathodes, suggesting more severe PEC performance degradation will be caused in the large‐scale full‐cell OHP dual photoelectrodes.

**Figure 5:**
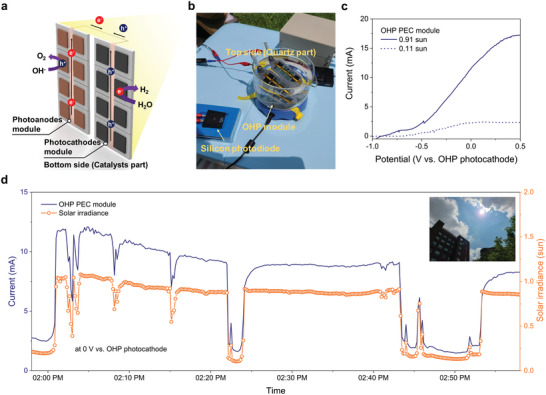
PEC performance of the OHP module for unassisted solar water splitting under natural sunlight. a) Schematic representation and b) photograph of the OHP module. c) Representative LSV curves of the OHP module with different solar irradiance of natural sunlight. d) Chronoamperometric measurement for real‐world verification of the OHP module without external bias as well as real‐time solar irradiance evaluation. The inset shows a photograph of the sky at 01:53 PM.

Considering these aspects, we attempted for the first time to minimize the reduction of PEC performance in a larger area by modularizing the OHP photoanodes and photocathodes, which exhibited the maximum PEC performance when they were characterized for each device with an active area of 0.25 cm^2^. As shown in **Figure** **5**a, the OHP module was constructed by connecting 8 devices of the OHP photoanodes and photocathodes in parallel, each of which was fabricated in one batch, with a total of 16 devices of the OHP photoelectrodes. The total active area of the constructed OHP module was 4 cm^2^. We investigated the PEC performance of the OHP module in 1 m KOH electrolytes using a two‐electrode configuration. For future practical application of the OHP module, we investigated the PEC performance of the OHP module under outdoor conditions as shown in the photograph in Figure 5b. Outdoor evaluation of the OHP module was conducted at the Gwangju Institute of Science and Technology, Gwangju, Republic of Korea (35°13′ N, 126°50′ E), under natural sunlight. The solar irradiance of natural sunlight was measured in real‐time (Figure 5b). As shown in the LSV curves (Figure 5c), the OHP module exhibited a photocurrent of 11.52 mA at the unassisted condition under 0.91 sun solar irradiance of natural sunlight. Although under a cloudy sky (0.11 sun solar irradiance), the OHP module can generate the photocurrent (2.21 mA) at the unassisted condition (Figure 5c). To investigate the PEC performance of the OHP module under real‐time change in the solar irradiance of natural sunlight, a chronoamperometric measurement of the OHP module was performed at the unassisted condition, as shown in Figure 5d. Simultaneously, the real‐time solar irradiance of natural sunlight was also measured (Figure 5d). At the initial measurement (01:58 PM), the OHP module exhibited a low photocurrent under low solar irradiance due to the clouds (Figure 5d). A photograph of the sky at 01:53 PM is shown in the inset of Figure 5d. After 02:01 PM, the photocurrent of the OHP module increased due to the increase in the solar irradiance as the clouds cleared (Figure 5d). The maximum photocurrent (12.09 mA) of the OHP module was achieved at 02:04 PM under a solar irradiance of 1.06 sun (Figure 5d). At 02:22 PM, a photocurrent decreased due to the reappearance of clouds. However, after 02:24 PM, the clouds cleared and the photocurrent recovered (Figure 5d). This recovery of the photocurrent suggests that low solar irradiance does not cause permanent degradation of the OHP module, and the potential of unassisted solar water splitting with the OHP module even with real‐time change in the solar irradiance of natural sunlight. However, after 02:24 PM, the delayed recovery of photocurrent was observed. It is difficult to reveal the delayed recovery of the photocurrent under natural sunlight in OHP module with certainty in this study, but we assume that this to be due to the degradation of the subcells included in the module. As shown in Figure 5d, the photocurrent responds immediately to solar irradiance before 2:24 PM. However, after that, the delayed recovery of photocurrent was observed. Therefore, it is assumed that the degradation of OHP devices has progressed under natural sunlight conditions. Before 2:24 PM, a slight photocurrent degradation was also occurred, suggesting degradation of subcells in PEC module. The O_2_ and H_2_ gases generated from the catalysts part of the OHP module at the unassisted condition under natural sunlight also demonstrate the feasibility of OHP module for scalable unassisted solar water splitting (Video S1, Supporting Information). From a practical standpoint of the OHP module, the separation of gas products is also required in the future. In general, gas products generated from the lab‐scale PEC devices are separated by ion‐exchange membranes. However, considering the practical standpoint, such ion‐exchange membranes not only impose additional complexity, but also cause degradation of PEC devices, which can be especially fatal issues in OHP‐based PEC devices during long‐term operation.^[^ ^44^ ^]^ Therefore, we suggest a membrane‐less architecture as an appropriate system for gas separation in OHP module. The photocurrent density under the unassisted condition of the OHP module was 2.88 mA cm^−2^, which was lower than that of the OHP dual photoelectrodes (8.75 mA cm^−2^).

Although we successfully demonstrated unassisted solar water splitting using the OHP module was successfully demonstrated, the photocurrent per unit area of the OHP module is still low compared with the optimized OHP dual photoelectrodes, each with an active area of 0.25 cm^2^. The relatively low photocurrent density obtained after modularization of the OHP dual photoelectrodes is mainly attributed to the inactive sub‐cells in the OHP module. Although all the OHP PV cells in the module were fabricated in one batch, the performance of each OHP PV cell is different, and some devices may exhibit poor performance or be inactive. In practice, the OHP photoanodes and photocathodes fabricated with inactive OHP PV cells do not exhibit any photoresponse (Figure S13a,b, Supporting Information). Note that the previously reported PEC module also suffered from the issue of inactive subcells.^[^ ^45^ ^]^ This inactivity of the OHP photoelectrodes is caused by macroscopic defects such as pinholes or cracks, which results in an undesirable shunt‐current leakage in the OHP PV cells. In particular, because OHP photoelectrodes include conductive contact materials, such as EGaIn and graphite epoxy, which can penetrate through pinholes or particles, shunt‐current leakage can occur more frequently as these conductive materials come into direct contact with the bottom electrodes. Nevertheless, in the case of a conventional single large PEC cell, degradation of just one photoelectrode damages the entire PEC system. On the other hand, the PEC module design is favorable in terms of practical and long‐term operation because the degradation of one photoelectrode damages only the subcells inside the module. To maximize the PEC performance of the OHP module, the following factors should be improved in the future: 1) preparation of OHP layers with minimal pinholes, 2) introduction of passivation layers in the OHP photoelectrodes to minimize the effects of pinholes, and 3) preselection of OHP photoelectrodes for the construction of OHP modules without inactive subcells. Although there are many improvements to be made, the OHP PEC module design provides an insight from a practical standpoint that the scalability, one of the challenges of OHP‐based PEC systems, can be overcome in a simple way. Hence, the OHP modules constructed with high reproducibility and pinhole‐free OHP PV cells will open new potential for large‐scale OHP‐based PEC systems.

## 3 Conclusion

In conclusion, we demonstrated the OHP module as a scalable design for OHP‐based PEC systems. Although the fabricated OHP‐dual photoelectrodes achieved an STH conversion efficiency of 10.4% at the unassisted condition, thus satisfying the requirements for commercial PEC systems, this high efficiency was only achieved on a small‐scale of less than 1 cm^2^. However, increasing the device area of the single OHP photoelectrodes causes severe degradation in the PEC performance due to an increase in the resistive loss. From this perspective, for the scaling‐up and commercialization of OHP‐based PEC systems, a breakthrough in the scalable design suitable for OHP photoelectrodes is urgently required. Thus, as a scalable design, we constructed and characterized an OHP module with an effective area of 4 cm^2^ by connecting the optimized OHP photoanodes and photocathodes. The OHP module constructed with a total of 16 devices of the OHP photoelectrodes achieved a photocurrent of 11.52 mA under 0.91 sun of natural sunlight without external bias. As a proof‐of‐concept, the successful demonstration of the OHP module under natural sunlight provides immense opportunities for further improving the scalability and commercialization of OHP‐based PEC systems.

## 4 Experimental Section

### Chemicals

The SnO_2_ nanoparticle colloid precursor (tin(IV) oxide, 15% in H_2_O colloidal dispersion) was purchased from Alfa Aesar. Lead(II) iodide (PbI_2_, 99.9985%) and 2PACz were purchased from Tokyo Chemical Industry. Methylammonium iodide (CH_3_NH_3_I) was purchased from GreatCell Solar. [6,6]‐phenyl‐C_61_‐butyric acid methyl ester (PCBM) was purchased from Nano‐C. 2,2′,7,7′‐tetrakis(*N,N*‐di‐4‐methoxyphenylamine)‐9,9′‐spirobifluorene (spiro‐OMeTAD) was purchased from Lumtec. PTAA was purchased from EM Index. PFN‐Br was purchased from 1‐Material. 4‐*tert*‐butylpyridine (*t*BP), bis(trifluoromethane)sulfonimide lithium salt (Li‐TFSI, 99.95%), tris(2‐(1*H*‐pyrazol‐1‐yl)‐4‐*tert*‐butylpyridine)‐cobalt(III) tri[bis(trifluoromethane)sulfonimide] (FK 209 Co(III) TFSI salt), bathocuproine (BCP), methanol (MeOH), acetonitrile (ACN, 99.8%), *N*,*N*‐dimethylformamide (DMF), dimethyl sulfoxide (DMSO), diethyl ether (DEE), toluene and chlorobenzene (CB), nickel(II) nitrate hexahydrate (Ni(NO_3_)_2_·6H_2_O, 98%), iron(II) sulfate heptahydrate (Fe(SO_4_)_2_·7H_2_O, 99%), nickel(II) sulfate hexahydrate (NiSO_4_·6H_2_O, 98%), sodium molybdate dihydrate (Na_2_MoO_4_·2H_2_O, 99%), sodium citrate dihydrate (Na_3_C_6_H_5_O_7_·2H_2_O, 99%), ammonium hydroxide solution (NH_4_OH, 28% in H_2_O), and EGaIn (99.99%) were purchased from Sigma‐Aldrich.

### Fabrication of OHP PV Cells

To fabricate the p–i–n OHP PV cells, indium‐doped tin oxide (ITO) substrates were cleaned with deionized (DI) water, acetone, and isopropanol for 20 min in each step in an ultrasonic bath. Subsequently, the cleaned substrates were stored in a hot furnace (85 °C in air) for a 1‐day drying process. In the case of PTAA/PFN‐Br HTLs for the p–i–n OHP PV cells, a 0.25 wt% of PTAA in toluene was deposited by spin‐coating at 5000 rpm for 30 s, followed by the deposition of 0.05 wt% PFN‐Br in MeOH at 5000 rpm for 30 s. In the case of 2PACz HTLs for the p–i–n OHP PV cells, 0.01 wt% 2PACz was deposited by spin‐coating at 3000 rpm for 30 s. Methylammonium lead triiodide (MAPbI_3_) solution was prepared at a molar ratio of 1.5:1.5 in DMF/DMSO (volume ratio of 9:1.1). The MAPbI_3_ film was spin‐coated at 5000 rpm for 30 s. Subsequently, 0.8 mL of DEE, an anti‐solvent, was added dropwise during the MAPbI_3_ film‐coating process in the initial 5 s. The substrate was transferred to a hot plate at 100 °C for 5 min. The PCBM (20 mg mL^−1^ in CB) layer was spin‐coated at 1500 rpm for 30 s to achieve high efficiency and thermal stability, and then the BCP layer was spin‐coated at 5000 rpm for 30 s. Finally, 200 nm of Cu electrode was deposited by thermal evaporation.

To fabricate the n–i–p OHP PV cells, a SnO_2_ colloid precursor was further diluted (that is, 1:6 w/w in H_2_O) and spin‐coated at 3,000 rpm for 30 s. The films were annealed at 100 °C for 1 h. A MAPbI_3_ solution was prepared at a molar ratio of 1.5:1.5 in DMF/DMSO (volume ratio of 9:1.1). The MAPbI_3_ film was spin‐coated at 5000 rpm for 30 s. Subsequently, 0.8 mL of DEE, an antisolvent, was added dropwise during the MAPbI_3_ film‐coating process in the initial 5 s. The substrate was transferred to a hot plate at 100 °C for 5 min. For deposition of the HTLs, a spiro‐OMeTAD solution in CB (90 mg mL^−1^) was prepared. In addition, 21 µL of Li‐TFSI in ACN (540 mg mL^−1^), 10 µL of FK 209 Co(III) TFSI in ACN (375 mg mL^−1^), and 39 µL of pure *t*BP were added to the spiro‐OMeTAD solution. The spiro‐OMeTAD solution was spin‐coated at 3,000 rpm for 30 s. Finally, 200 nm of Au electrode was deposited by thermal evaporation.

### Device Characterization of the OHP PV Cells

The *J*−*V* curves were measured using a Keithley 238 source measure unit (Keithley Instruments, Inc.) under AM 1.5 G solar simulated illumination emitted at 100 mW cm^−2^ from a solar simulator (Newport 750). The intensity of the Xe lamp (150 W Oriel) was calibrated using a National Renewable Energy Laboratory (NREL)‐calibrated KG5 filtered silicon reference cell. For accurate measurement, the *J*−*V* characteristics were cross‐checked by integrating the EQE from a solar cell spectral response/QE/EQE measurement system (PV Measurements, Inc.). For the evaluation of stability, the nonencapsulated OHP PV cells were kept under dark and ambient condition (in air). The *J*−*V* characterizations for the OHP cells were performed under AM 1.5 G illumination in a N_2_ atmosphere.

### Integration of Catalysts with Encapsulation Layers

NiFe LDH and NiMo were electrodeposited on Ni foils using previously reported methods with slight modification.^[^ ^31^, ^32^ ^]^ Prior to electrodeposition, the Ni foils (0.05 mm thickness, 99%, Alfa Aesar) were cleaned with acetone, methanol, isopropanol, and DI water. Before the electrodeposition, cleaned Ni foils were immersed in hydrochloric acid (10% in H_2_O) for 5 min and washed with DI water. NiFe LDH was electrodeposited using a three‐electrode configuration with Ni foil, Pt mesh, and Ag/AgCl (saturated KCl) as the working, counter, and reference electrodes, respectively. An aqueous solution of 0.075 m of Ni(NO_3_)_2_·6H_2_O and 0.025 m of Fe(SO_4_)_2_·7H_2_O was used for electrodeposition of NiFe LDH. To prevent the oxidation of Fe^2+^, the aqueous solution was purged with Ar gas. The electrodeposition was performed at a potential of −1.0 V versus Ag/AgCl for 90 s. NiMo was electrodeposited using a three‐electrode configuration with Ni foil, Pt mesh, and Ag/AgCl (saturated KCl) as the working, counter, and reference electrodes, respectively. For electrodeposition of NiMo, a constant current density of −80 mA cm^−2^ for 45 min was used in an aqueous solution of 0.03 m of NiSO_4_·6H_2_O, 0.02 m of Na_2_MoO_4_·2H_2_O, and 0.03 m of Na_3_C_6_H_5_O_7_·2H_2_O. The pH of the NiMo precursor solution was adjusted to be 10.5 with NH_4_OH. After electrodeposition, both the NiFe LDH and NiMo films were washed with ethanol and DI water, followed by dried at 60 °C in an oven.

### Materials Characterization of the Catalyst‐Intergrated Encapsulation Layers

The morphological characteristics of NiFe LDH and NiMo were analyzed by using FESEM (Verios 5 UC, Thermo Fisher Scientific) and TEM (Tecnai G2 F20, FEI). The structural characteristics of NiFe LDH and NiMo were analyzed by using XRD patterns obtained by X‐ray diffractometer (SmartLab, Rigaku). The chemical states of NiFe LDH and NiMo were analyzed by XPS (NEXSA, Thermo Fisher Scientific).

### Fabrication of the OHP Dual Photoelectrodes and Modularization

To fabricate the OHP photoanodes, the prepared n–i–p OHP PV cells and NiFe LDH/Ni foil encapsulation layers were combined with graphite epoxy. The graphite epoxy was prepared by a mixture of graphite powder and epoxy resin (mass ratio of 3:4) as previously reported.^[^ ^19^ ^]^ To fabricate the OHP photocathodes, the prepared p–i–n OHP PV cells and NiMo/Ni foil encapsulation layers were combined with EGaIn.^[^ ^27^, ^28^ ^]^ Subsequently, Cu wire and ITO electrodes were combined with Ag paste. After the Ag paste was dried, both the fabricated OHP photoelectrodes were sealed by epoxy resin to prevent the penetration of water molecules into the OHP layers. The active area of each OHP photoelectrode was defined to be 0.25 cm^2^.

For modularization of the OHP photoelectrodes, each of the 8 devices of the OHP photoanodes and photocathodes (total 16 devices) fabricated in one batch were connected in parallel on quartz substrate. As in the fabrication of the OHP photoelectrodes, Cu wire and ITO electrodes were combined with Ag paste. After the Ag paste was dried, the connected OHP photoelectrode arrays were sealed with epoxy resin.

### Measurements of the EC and PEC Performance

EC and PEC measurements were conducted with a potentiostat (Nstat, Ivium Technologies). In this study, all measured potentials (versus Ag/AgCl) in this study were converted to vs. RHE using the following Nernst equation

(1) $$E_{RHE}=E_{Ag/AgCl}+0.0592\times pH+0.197$$

where *E*_RHE_ is the converted potential (versus RHE) and *E*_Ag/AgCl_ is the measured potential (versus Ag/AgCl).

The OER and HER catalytic activities of the encapsulation layers were measured in 1 m KOH electrolytes. A standard three‐electrode configuration was used with catalyst‐integrated or bare Ni foil, graphite rod, and Ag/AgCl (saturated KCl) as the working, counter, and reference electrodes, respectively. The CV curves of the catalyst‐integrated or bare Ni foil were measured at a scan rate of 10 mV s^−1^ without *iR* compensation.

The PEC performance of the OHP photoelectrodes was measured in 1 m KOH electrolytes using a standard three‐electrode configuration. For measurements of the OHP photoanode, the NiFe LDH/Ni foil/OHP photoanode, graphite rod, and Ag/AgCl (saturated KCl) were used as the working, counter, and reference electrodes, respectively. For the OHP photocathode, the NiMo/Ni foil/OHP photocathode, graphite rod, and Ag/AgCl (saturated KCl) were used as the working, counter, and reference electrodes, respectively. To evaluate the PEC performance of the OHP photoelectrodes, the backside of the OHP photoelectrodes was illuminated under AM 1.5 G (100 mW cm^−2^) with a 150 W Xe lamp (Model 10500, ABET Technology). The CV curves of the OHP photoelectrodes were measured at a scan rate of 10 mV s^−1^.

The PEC performance of the OHP dual photoelectrodes for unassisted solar water splitting was measured in 1 M KOH electrolytes by using a two‐electrode configuration, with the OHP photoanodes and photocathodes as the working and counter electrodes, respectively. Two solar simulators were used to evaluate the PEC performance of the OHP dual photoelectrodes. Meanwhile, the PEC performance of the OHP module was measured under natural sunlight. The LSV curves of the OHP dual photoelectrodes were measured at a scan rate of 10 mV s^−1^. The PEC performance of the OHP module was measured in 1 m KOH electrolytes by using a two‐electrode configuration with the OHP photoanodes and photocathodes connected in parallel as the working and counter electrodes, respectively. The LSV curves of the OHP module were measured at a scan rate of 10 mV s^−1^. Outdoor evaluation of the OHP module to measure PEC performance was conducted at the Gwangju Institute of Science and Technology, Gwangju, Republic of Korea, (35°13′ N, 126°50′ E), under natural sunlight. Solar irradiance of natural sunlight was measured in real‐time with a silicon photodiode (BS‐500BK, Bunkoukeiki).

The *η*_F_ was determined by measuring the amount of O_2_ and H_2_ gases generated at the OHP photoanode and photocathode, respectively, through gas chromatography (6500 GC, Young Lin Instrument Co. Ltd.).

The STH conversion efficiency for OHP dual photoelectrodes for unassisted solar water splitting was evaluated by using the following equation^[^ ^46^ ^]^

(2) $$STH=\frac{J_{ph}\times 1.23\times \eta_{F}}{P}\times 100\%$$

## Conflict of Interest

The authors declare no conflict of interest.

## Supporting information

Supporting Information

Supplemental Video 1

## Data Availability

The data that support the findings of this study are available on request from the corresponding author. The data are not publicly available due to privacy or ethical restrictions.
