# Supplementary material for: Organometal Halide Perovskite‐Based Photoelectrochemical Module Systems for Scalable Unassisted Solar Water Splitting
Source: Adv Sci (Weinh). 2023 Sep 26;10(33):2303106. doi: 10.1002/advs.202303106 (PMC10667810; doi:10.1002/advs.202303106)
Supplement: Supplementary file 1 — Supporting Information [file ADVS-10-2303106-s002.pdf]

ADVANCED SCIENCE
Open Access

Supporting Information

for *Adv. Sci.*, DOI 10.1002/advs.202303106

Organometal Halide Perovskite-Based Photoelectrochemical Module Systems for Scalable Unassisted Solar Water Splitting

Hojoong Choi, Sehun Seo, Chang Jae Yoon, Jae-Bin Ahn, Chan-Sol Kim, Yoonsung Jung, Yejoon Kim, Francesca M. Toma, Heejoo Kim and Sanghan Lee**

Supporting Information

Organometal Halide Perovskite-Based Photoelectrochemical Module Systems for Scalable Unassisted Solar Water Splitting

Hojoong Choi,[1,†] Sehun Seo,[2,3,4,†] Chang Jae Yoon,[5] Jae-Bin Ahn,[5] Chan-Sol Kim,[5] Yoonsung Jung,[1] Yejoon Kim,[1] Francesca M. Toma,[2,3,4] Heejoo Kim,[5,6,] and Sanghan Lee.[1,7,*]*

[1] School of Materials Science and Engineering, Gwangju Institute of Science and Technology, Gwangju 61005, Republic of Korea

[2] Chemical Sciences Division, Lawrence Berkeley National Laboratory, 1 Cyclotron Road, Berkeley, CA 94720, USA

[3] Liquid Sunlight Alliance, Lawrence Berkeley National Laboratory, 1 Cyclotron Road, Berkeley, CA 94720, USA

[4] Institute of Functional Materials for Sustainability, Helmholtz-Zentrum Hereon, Kantstraße 55, 14513 Teltow, Germany

[5] Research Institute for Solar and Sustainable Energies, Gwangju Institute of Science and Technology, Gwangju 61005, Republic of Korea

[6] Graduate School of Energy Convergence, Institute of Integrated Technology, Gwangju Institute of Science and Technology, Gwangju 61005, Republic of Korea

[7] Research Center for Innovative Energy and Carbon Optimized Synthesis for Chemicals (Inn-ECOSysChem), Gwangju Institute of Science and Technology, Gwangju 61005, Republic Korea

[†] These authors contributed equally to this work.

[*] E-mail address: heejook@gist.ac.kr (H.K.), sanghan@gist.ac.kr (S.L.)

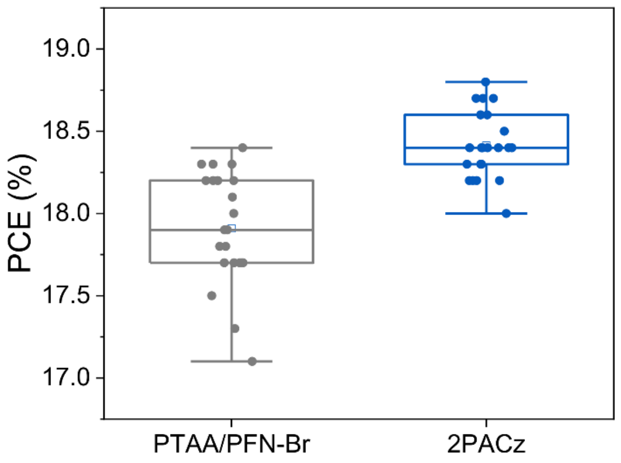

Figure S1. Statistical power conversion efficiency (PCE) distribution of the p-i-n organometal halide perovskite (OHP) photovoltaic (PV) cells with different hole transport layers (HTLs).

Table S1. Average and maximum PCE of the p-i-n OHP PV cells with different HTLs.

	PCE_{avg} (%)	PCE_{max} (%)
PTAA/PFN-Br	17.91 ± 0.35	18.4
2PACz	18.41 ± 0.2	18.8

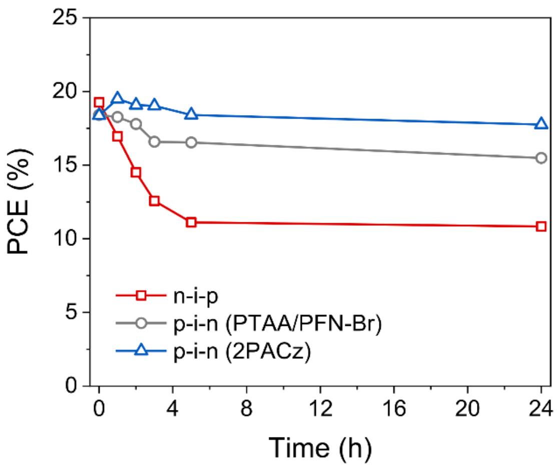

Figure S2. Stability of the OHP PV cells (after kept under 25 °C/85% relative humidity).

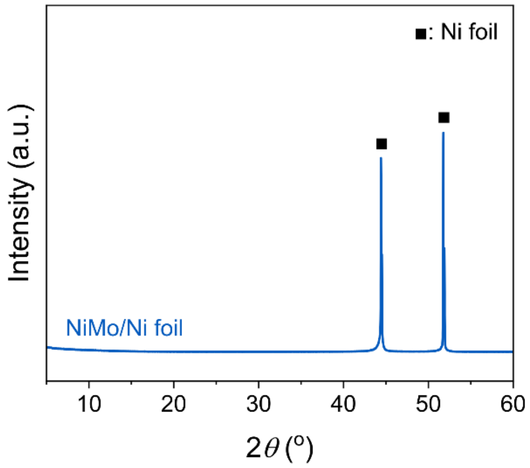

Figure S3. X-ray diffraction (XRD) pattern of NiMo/Ni foil.

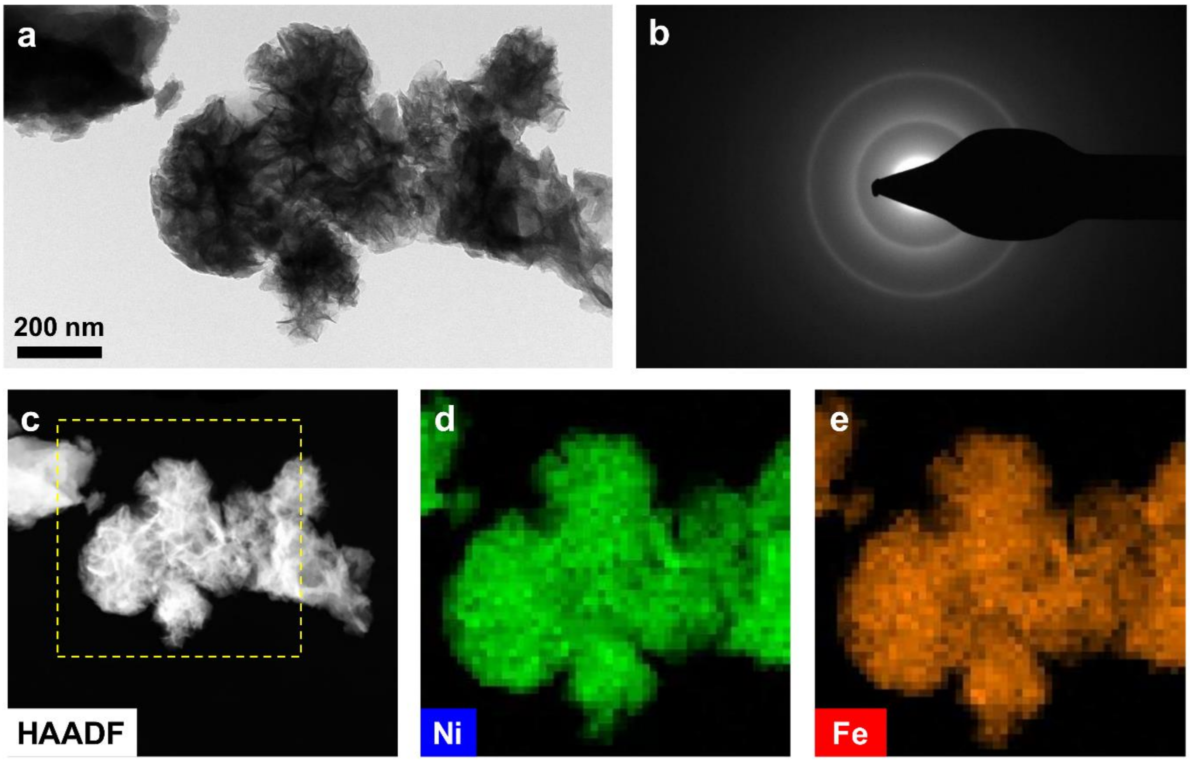

Figure S4. Morphological and structural characteristics of the NiFe layered double hydroxide (LDH). a) Transmission electron microscopy (TEM) image, b) selective area electron diffraction (SAED) pattern, and c) high-angle annular dark field scanning-TEM (HAADF-STEM) image of the NiFe LDH. d,e) Energy-dispersive spectroscopy (EDS) mapping results of the NiFe LDH obtained from a yellow-dotted region in corresponding HAADF-STEM image.

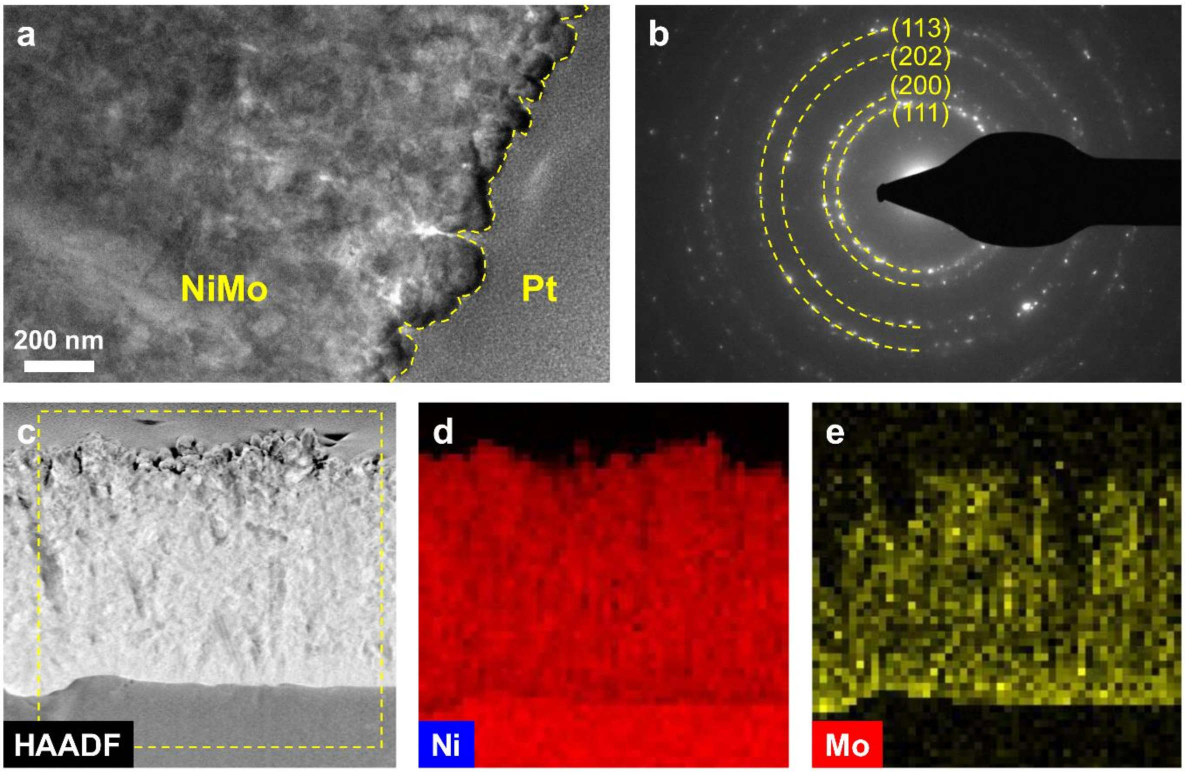

Figure S5. Morphological and structural characteristics of the NiMo. a) TEM image, b) SAED pattern, and c) HAADF-STEM image of the NiMo. d,e) EDS mapping results of the NiMo obtained from a yellow-dotted region in corresponding HAADF-STEM image.

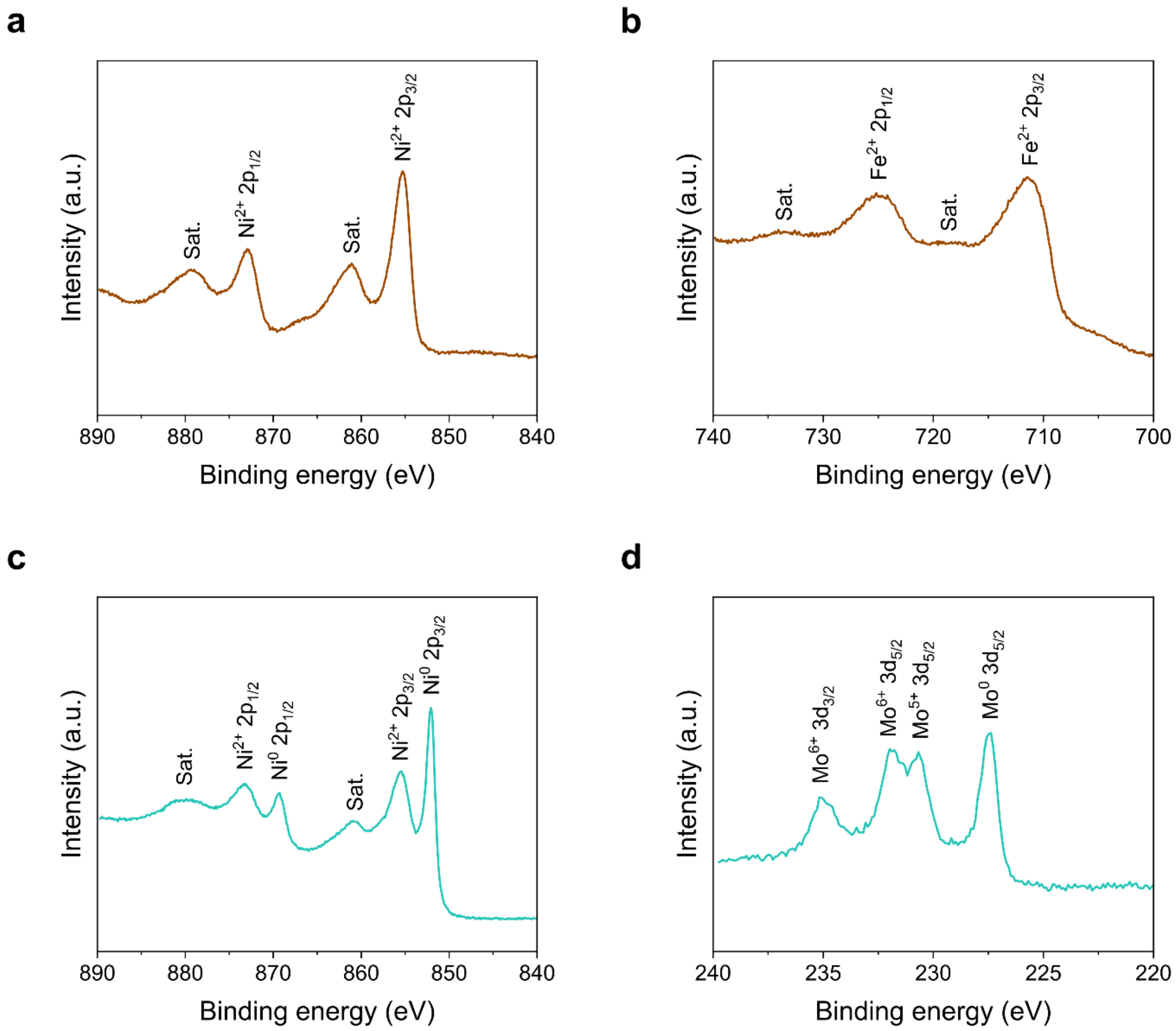

Figure S6. High-resolution X-ray photoelectron spectroscopy (XPS) spectra of a) Ni 2p and b) Fe 2p for NiFe LDH/Ni foil, and c) Ni 2p and d) Mo 3d for NiMo/Ni foil.

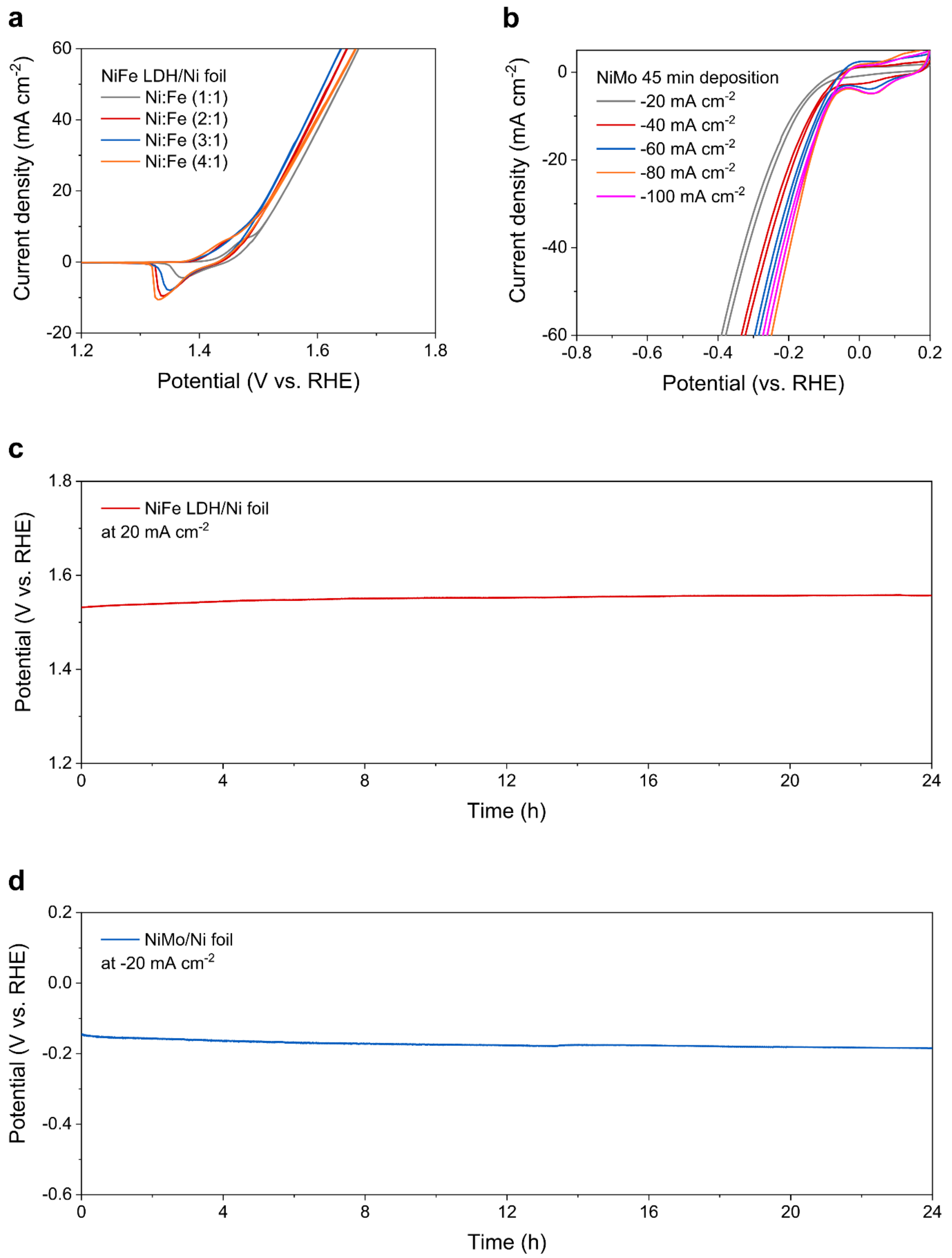

Figure S7. Cyclic voltammetry (CV) curves of the a) NiFe LDH/Ni foils with different ratios of Ni:Fe precursors and b) NiMo/Ni foils with different current densities during electrodeposition. Chronopotentiometric measurements of the c) NiFe LDH/Ni foil at 20 mA cm^{-2} and d) NiMo/Ni foil at −20 mA cm^{-2}.

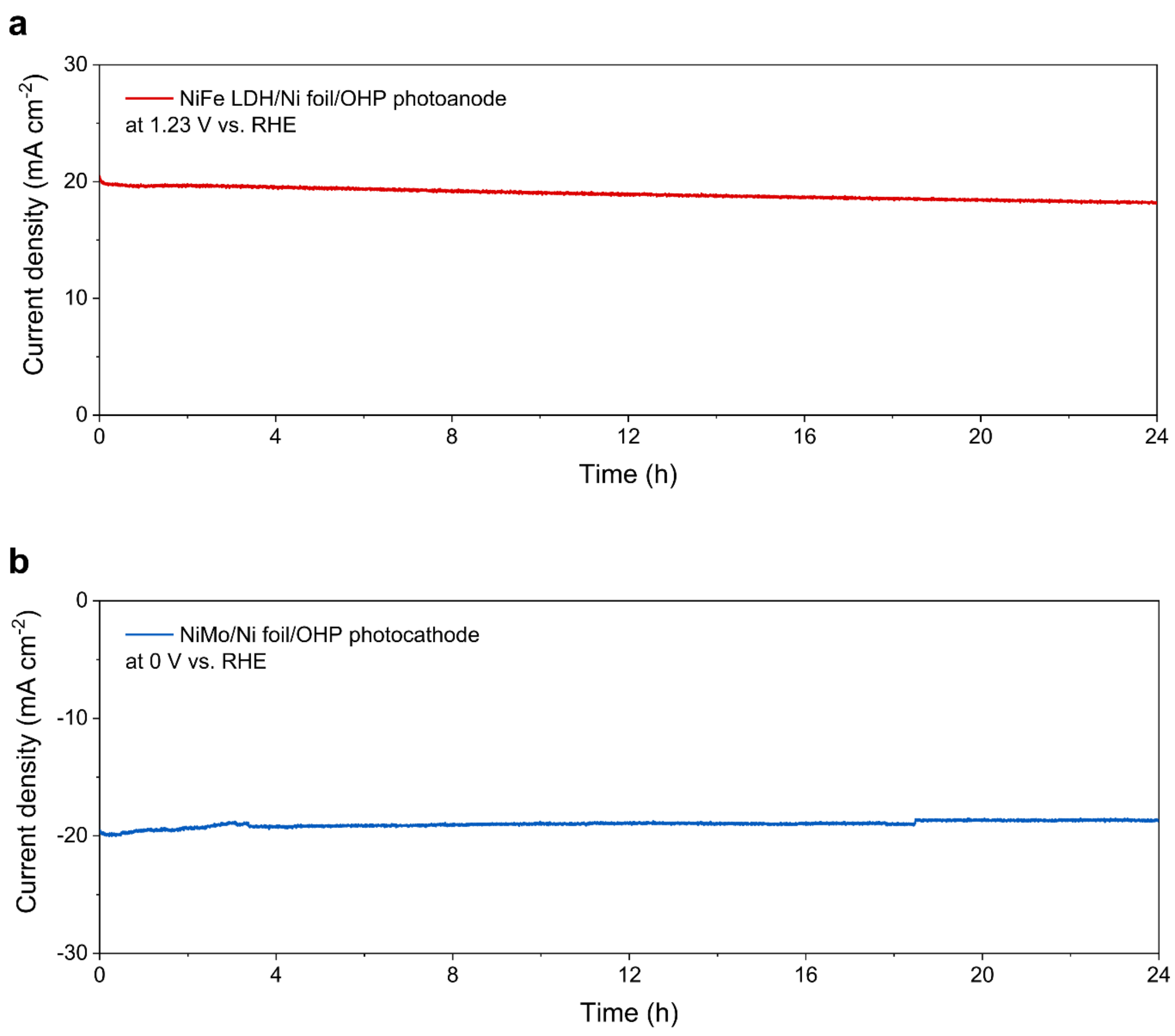

Figure S8. Chronoamperometric measurements of the a) NiFe LDH/Ni foil/OHP photoanode and b) NiMo/Ni foil/OHP photocathode under continuous illumination.

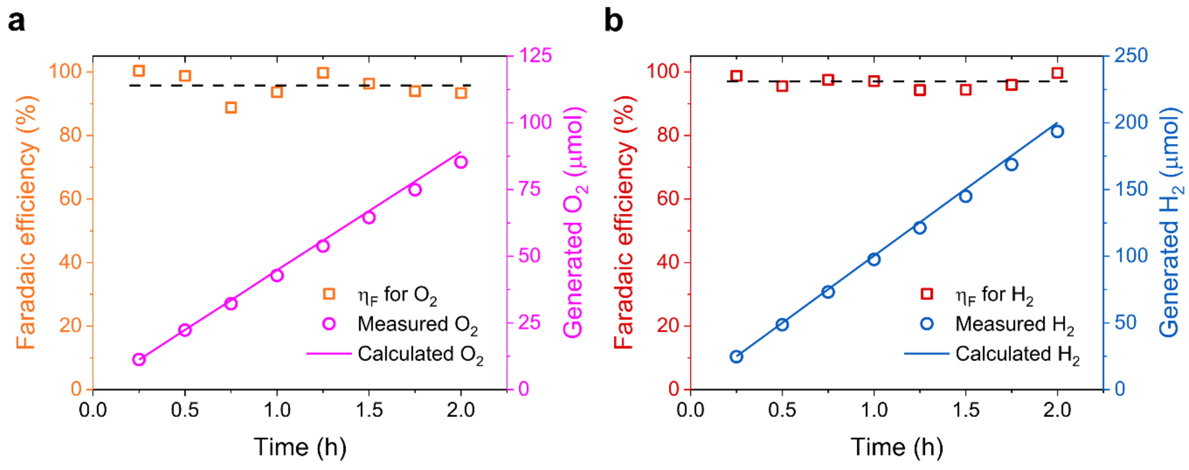

Figure S9. Generated a) O_2 and b) H_2 gases at the OHP photoanode and photocathode, respectively. Corresponding Faradaic efficiency (η_F) for a) O_2 and b) H_2 evolution.

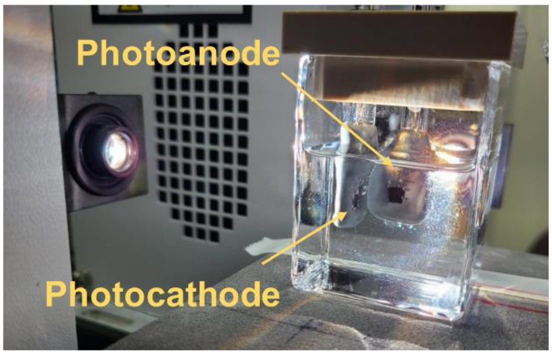

Figure S10. Photograph of the configuration of the OHP dual photoelectrodes during the photoelectrochemical measurements.

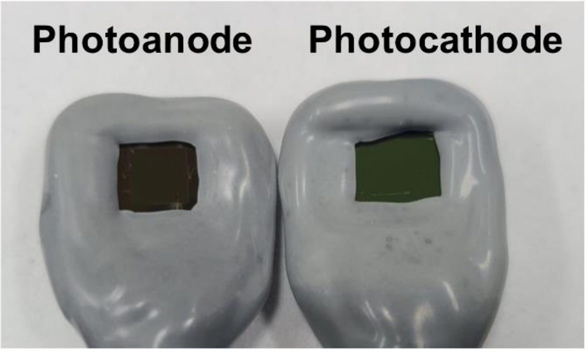

Figure S11. Photograph of the OHP dual photoelectrodes after the chronoamperometric measurements.

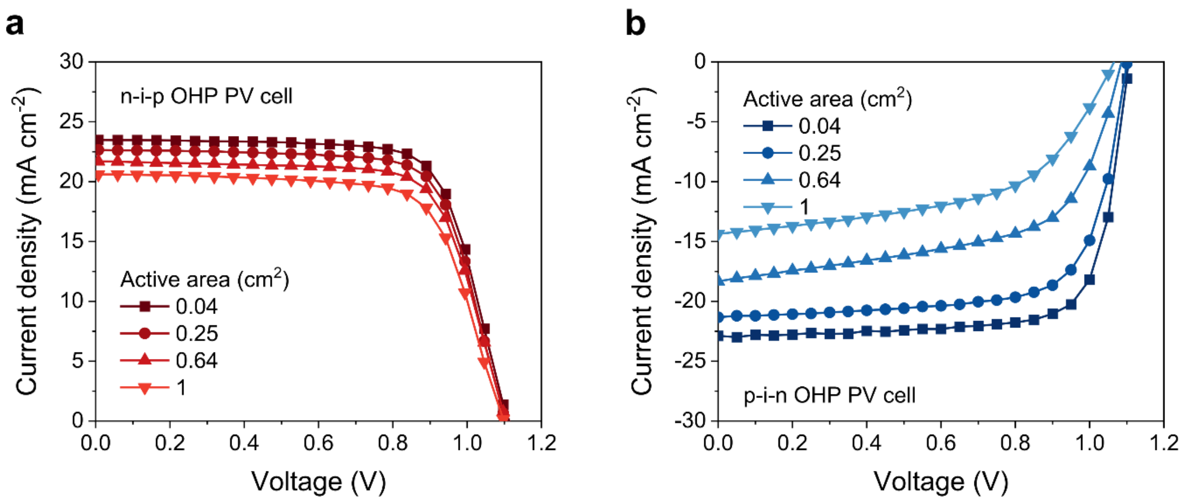

Figure S12. Current density–voltage (*J*–*V*) curves of the a) n-i-p and b) p-i-n OHP PV cells with various active areas.

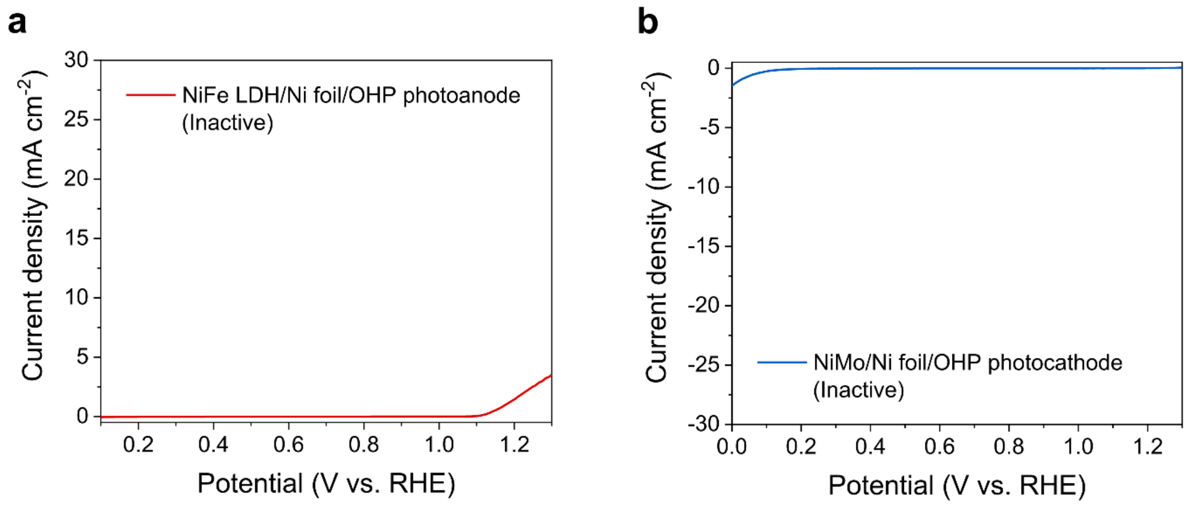

Figure S13. Linear sweep voltammetry (LSV) curves of the inactive a) NiFe LDH/Ni foil/OHP photoanode and b) NiMo/Ni foil/OHP photocathode under illumination.
